# PMMA/ABS/CoCl_2_ Composites for Pharmaceutical Applications: Thermal, Antimicrobial, Antibiofilm, and Antioxidant Studies

**DOI:** 10.3390/molecules27227669

**Published:** 2022-11-08

**Authors:** Muhammad Abid Zia, Muhammad Kaleem Khosa, Awal Noor, Sadaf Qayyum, Muhammad Shabbir Shakir

**Affiliations:** 1 Department of Chemistry, Government College University Faisalabad, Faisalabad 36000, Pakistan; 2 Department of Basic Sciences, Preparatory Year Deanship, King Faisal University, Al-Hassa 31982, Saudi Arabia

**Keywords:** biofilms, bacteria, degradation, inorganic filler

## Abstract

In this study, PMMA/ABS/CoCl_2_ ternary composite films were fabricated by the solution casting technique. The different weight ratios of cobalt chloride (≤10 wt) were incorporated into the PMMA/ABS blend (80:20). The chemical structure and thermal properties of the synthesized composites were assessed by FT-IR, TGA, and XRD. The biological properties of ternary composites, such as in vitro antibacterial activity and antioxidant capacity, were investigated. The enhanced thermal stability and promising antibacterial, selective antibiofilm, and potential antioxidant properties of PMMA/ABS/cobalt chloride composites demonstrated that they can be used for high-quality plastics and in many pharmaceutical applications.

## 1. Introduction

Composite materials are considered superior materials to other well-known structural materials due to strength, hardness, high temperature resistance, and fatigue strength. The poly acrylate hybrid materials as a matrix have received much attention for their extraordinary thermo-mechanical properties. Such new materials with increased properties and applications can be synthesized by a polymer-blending method [1,2,3]. Previous studies have shown that combining two or more polymers of various structures and compositions can produce products with varying physical, biological, and chemical properties. These materials are broadly utilized in an assortment of different biomedical fields such as dentistry, prosthetics, and helpful medication [4]. Polymethylmethacrylate (PMMA) is a class of polyacrylates that has been widely used for the preparation of special devices for electronic industries due to its high optical transparency. However, it has low mechanical strength, especially when it scratches the surface. It has limited applications due to its stress cracking, weak heat resistance, and limited solubility in organic solvents. High flexibility, simple methods of preparation, acceptable biocompatibility, and physical and chemical stability have led to its widespread use in healthcare [5]. Acrylonitrile–butadiene–styrene (ABS) resin is another type of co-polymer with three monomers, namely, acrylonitrile, butadiene, and styrene. Each component of ABS contributes its properties to the end product. Acrylonitrile–butadiene–styrene (ABS) is extensively used in household appliances and automotive industries because of its low moisture absorption ability and good mechanical properties [6,7]. ABS plastics are also thought to be a promising antibacterial agent because they are frequently used in areas where bacteria can grow easily, such as refrigerators, air conditioners, and toilet lids [8]. In the last few years, metal oxide and chloride fillers such as ZnO, MnO_2_, and CoCl_2_ and many nano inorganics were mixed with polymers and attracted much attention due to their good carrier mobility in improving toughness, promising antibacterial and UV protection properties even in an amorphous state [9,10,11]. Although metals have long been used as antibacterial agents in commercial products, some argue that they react slowly, and that the excessive use of heavy metals can lead to serious environmental pollution [12,13]. Further, the antibacterial activity will be poorer if the antibacterial coating is ruptured [14]. However, the use of antibacterial agents in PMMA and ABS may increase the chance of resistance of bacterial against drug and toxicity to humans and the environment [15]. To overcome such issues, a new methodology is highly needed. Keeping this in mind, polymethyl methacrylate (PMMA) and acrylonitrile butadiene styrene (ABS) were selected as a polymer matrix, and CoCl_2_ (≤10 wt) was used as a reinforcement material. The influence of cobalt chloride on structural and thermomechanical stability as well as the antibacterial and antioxidant activities of PMMA/ABS blends was explored.

## 2. Results and Discussion

### 2.1. Structural Analysis by FT-IR

**Figure 1:**
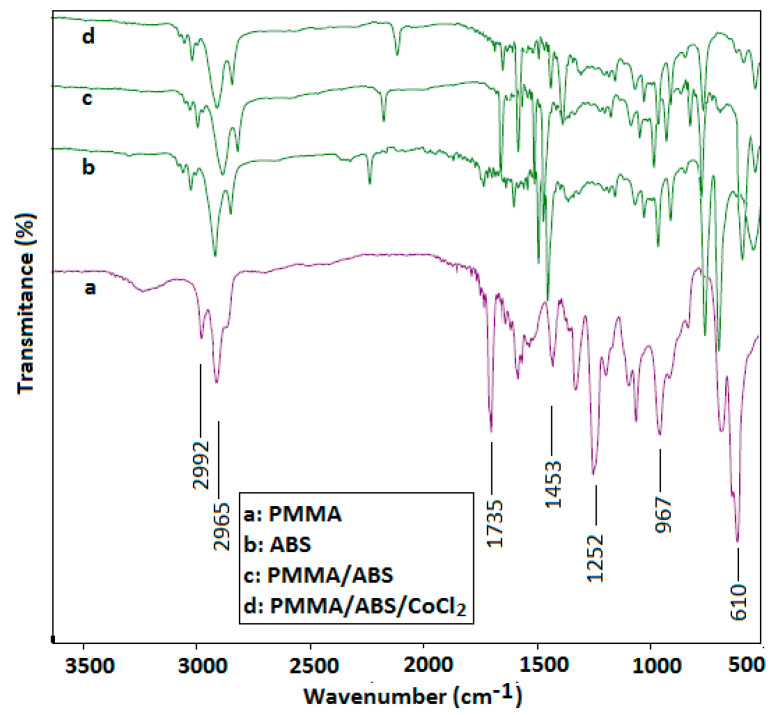
FT-IR of PMMA/ABS blend (80/20) and PMMA/ABS/CoCl_2_.

The FT-IR absorption data presented in Figure 1 show the different absorption bands corresponding to PMMA are sharp bands at 2965 cm^−1^ and 2992 cm^−1^ of ester methyl, while the band at 1735 cm^−1^ was due to C-O stretching vibration. Pure ABS has a characteristic absorption band at 698 cm^−1^ of –CH_2_ rocking vibration, whereas the band at 2235 cm^−1^ was because of the -R-C≡N group, being absent in the PMMA and ABS blend. Homogenous mixing of PMMA and ABS was confirmed by the shifting of the sharp band at 1735 cm^−1^ in pure PMMA to 1632 cm^−1^. As a result of the interaction between PMMA/ABS and CoCl_2_, the band at 1445 cm^−1^ disappeared, and a new sharp band at 1510 cm^−1^ appeared, shifting the acrylonitrile peak towards 2190 cm^−1^ [16,17,18].

### 2.2. X-ray Diffraction

**Figure 2:**
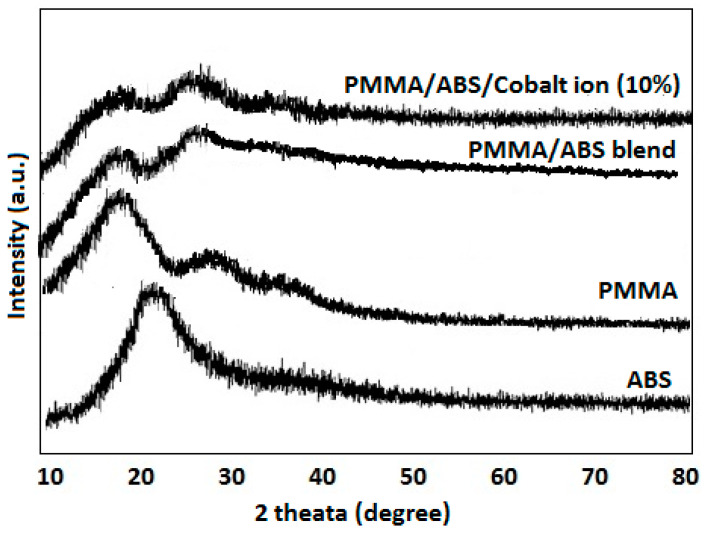
XRD pattern of PMMA/ABS/CoCl_2_ composite.

The degree of crystallinity of the PMMA/ABS blend and PMMA/ABS with CoCl_2_ (2.5, 5, 7.5, and 10%) was investigated using powder XRD. The X-ray diffractogram of pure PMMA showed three broad peaks at 18°, 30°, and 39°, while ABS displayed a characteristic broad peak at 2θ of 20° with a shoulder at 2 of 9°, indicating the copolymer’s amorphous nature, whereas when CoCl_2_ was incorporated into the polymer matrix, the same spectrum was recorded, without any obvious peak at 9°. Further, by increasing the concentration of CoCl_2_, there was a decrease in intensity and a broadening of the halo area was observed. Such results as presented in Figure 2 indicate that the cobalt chloride changed the structure of the polymeric matrices due to good dispersion in the polymeric matrices [19,20].

### 2.3. Thermal Analysis (TGA)

**Figure 3:**
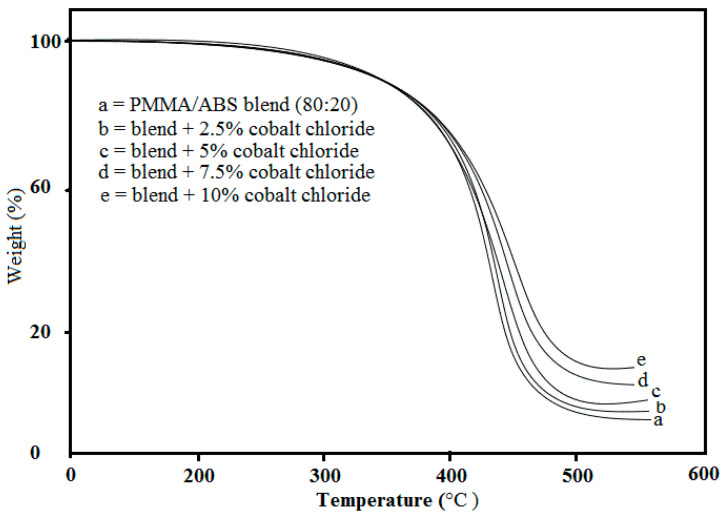
TGA thermogram of prepared PMMA/ABS/CoCl_2_ composite.

The thermal properties of PMMA/ABS blend with varying contents of cobalt chloride (2.5, 5, 7.5, and 10% *w*/*w*) was assessed up to 700 °C in terms of (*T*_0_), 50% weight loss (*T*_50_), (*T_max_*) maximum decomposition temperature, and ash contents at 700 °C. The thermograms are shown in Figure 3. The thermogram curves show that there was a single-stage decomposition in the PMMA/ABS matrix only because the metal salt had a high melting temperature. The initial volatilization started at around 350 °C with a sharp loss of weight that occurred at 400 °C. From the results, it was discovered that increasing the content of CoCl_2_ gradually increased the thermal stability.

### 2.4. Mechanical Properties

The effect of cobalt chloride on the mechanical properties of a PMMA/ABS blend and PMMA/ABS/CoCl_2_ was studied, and the data are shown in Table 1. The results revealed that the addition of different weight ratios of cobalt chloride increased the mechanical properties of the PMMA/ABS/CoCl_2_ composite. The impact strength and elongation at break increased significantly as the cobalt chloride content of the PMMA/ABS blend increased, whereas the tensile strength and elastic modulus decreased monotonously [21].

### 2.5. Antibacterial Activity

PMMA/ABS/CoCl_2_ with varying cobalt chloride concentrations showed promising antibacterial activity against all tested pathogenic strains. The results of the synthesized composite materials are presented in Table 2. According to the findings, the tested bacterial strains were more sensitive to 10% cobalt chloride with an inhibition zone of 20–31 mm. As the concentration of cobalt chloride in the PMMA/ABS blend increased, Gram-positive bacteria became more resistant. Among the pathogenic strains tested, *Staphylococcus aureus* had the highest sensitivity to 10% CoCl_2_ in the PMMA/ABS blend with a 31 mm zone of inhibition at 5 µL, while *Escherichia coli* had the lowest sensitivity with a 20 mm zone of inhibition, showing that Gram-negative pathogens appear to be resistant to metal salts. The sensitivity of Gram-positive and Gram-negative bacteria differs because of different morphologies of the tested strains, which results in resistance to the addition of composites loaded with 10% metal salts through the bacterial cell walls. These findings are also consistent with previous research [22]. The size of the metal, the uniform mixing of metal salts in polymeric matrix, and the chemical composition of composites may all have a greater impact on their bioactivity [23,24,25,26]. Furthermore, the higher the concentration of metal salt in the polymeric matrix, the more effective it is at inhibiting bacterial growth [27].

### 2.6. Antibiofilm Activity

**Figure 4:**
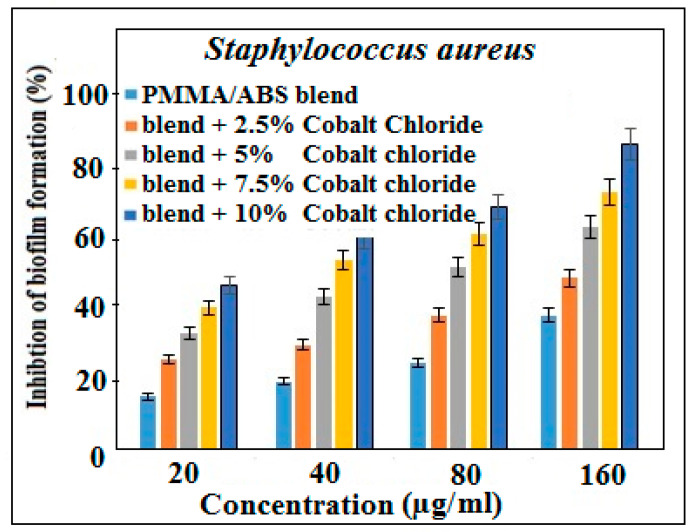
Biofilm inhibition activity of PMMA/ABS and PMMA/ABS/different concentrations of CoCl_2_ in DMSO, *p* < 0.05 vs. control.

**Figure 5:**
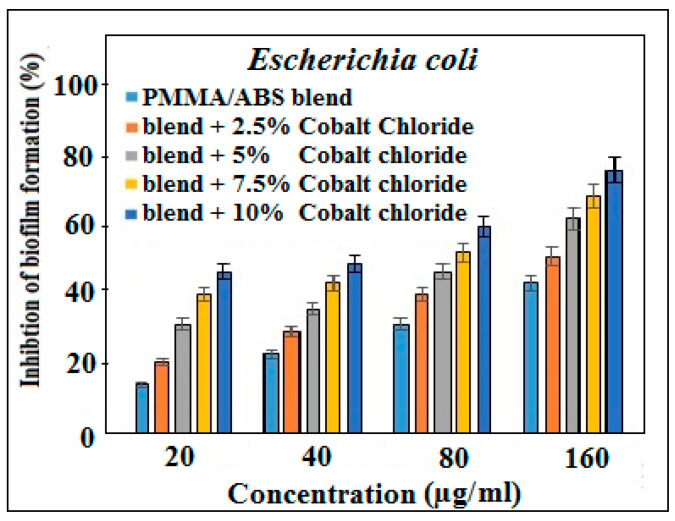
Biofilm inhibition activity of PMMA/ABS and PMMA/ABS/different concentrations of CoCl_2_ in DMSO, *p* < 0.05 vs. control.

The biofilm reduction ability of the PMMA/ABS blend and PMMA/ABS/CoCl_2_ was investigated against selective pathogenic strains, showing the highest and least resistivity against *S. aureus* and *E. coli* as model microorganisms, respectively. PMMA/ABS contains varying amounts of cobalt chloride, showing promising activity as compared to pure PMMA/ABS. The biofilm reduction activity was determined by the treatment of the tested bacterial strains with a PMMA/ABS blend and a PMMA/ABS/CoCl_2_ mixture that revealed that the inhibition effect of cobalt chloride is concentration dependent (Figure 4 and Figure 5). At 160 µg/mL concentrations of composite materials, the percentages of biofilm reduction for *S. aureus* and *E. coli* were 83 and 71, respectively. These results show that cobalt-chloride-containing composite materials inhibit biofilms more effectively than a PMMA/ABS blend.

## 3. Antioxidant Activity

The free radical inhibition features of PMMA/ABS films (80:20) with varying contents of cobalt chloride were determined using DPPH**^·^**, ABTH**^+^**, and FRAP assays, and the results are shown in Table 3. The free radical scavenging activity by DPPH· was of 55%, 59%, 66%, 78%, and 89%; by ABTH**^·^**^+^scavenging assay was of 27%, 30%, 44%, 58%, and 65%; and by FRAP assay was of 0.15%, 0.19%, 0.64%, 0.78%, and 0.94%. The results revealed that free radical inhibition properties are concentration dependent and increased as concentration of CoCl_2_ increased.

## 4. Materials and Method

### 4.1. Chemicals

Polymethylmethacrylate (PMMA) with a molecular weight of 100,000 and acrylonitrile–butadiene–styrene (ABS) were purchased from Sigma-Aldrich. Cobalt chloride (CoCl_2_), tetrahydrofuran (THF), 1,1-diphenyl-2-picrylhydrazyl (DPPH), tripyridyl-s-triazine (TPTZ), 2,2-azino-bis-(3-ethylbenzothiazoline-6-sulfonic acid (ABTH), and dimethylsulfoxid (DMSO) were purchased from Aldrich. Solvents were dried before they were used. Culture media and standard antibiotic discs were purchased from Oxoid Ltd. (Hampshire, UK).

### 4.2. Instruments

The prepared composite materials were characterized by using a variety of techniques. FT-IR spectra were recorded by an infrared spectrometer (Bruker tensor 27 FT-IR spectrometer) in a wavelength range of 4000–400 cm^−1^ to confirm different functional groups of synthesized PMMA/ABS and PMMA/ABS/CoCl_2_. For thermo-gravimetric analyses, a Perkin Elmer TGA-7 instrument was used under nitrogen at 50 mL and 10 °C min^−1^ up to 700 °C. Thermal stability of the composites was assessed in terms of *T*_0_, *T*_50_, *T_max_*, and ash content. The structural properties, cryatalanity, and pattern of composites were investigated using an X-ray diffraction 3040/60 X’ Pert PRO diffractrometer.

### 4.3. Preparation of PMMS/ABS/CoCl_2_ Composite Films

The solution casting technique was used to fabricate the PMMS/ABS/CoCl_2_ composite films [28]. Generally, 0.8 g PMMA and 0.2 g ABS solutions in (25 mL THF) were prepared separately. The polymer solutions were then mixed and stirred at 40 °C until we obtained a homogenous solution of a PMMA/ABS blend with a 80/20 weight ratio. CoCl_2_ solution in 15 mL THF with weight ratios of 2.5, 5.0, 7.5, and 10% was added dropwise to the PMMA/ABS blend solution and was stirred vigorously at room temperature until complete miscibility. Following that, a thick solution of PMMA/ABS/CoCl_2_ was cast onto Teflon boats and dried overnight to form uniform and transparent films with thicknesses of 40–50 µm. The films were kept overnight in an oven at 60 °C, then placed in vacuum desiccators for complete removal of solvent.

### 4.4. Antibacterial Methodology

The ternary composites (PMMA/ABS/CoCl_2_) were used to evaluate the antimicrobial activity by the disc-diffusion method using different bacterial strains, including *Staphylococcus aureus*, *Bacillus subtilis*, *Streptococcus pyogenes*, *Pseudomonas aeruginosa*, *Escherichia coli*, and *Salmonella typhi* [29,30,31]. About 100 µL of pathogenic strains was cultured in agar-agar nutrient medium at 37 °C for about 24 h. A broth culture of approximately 10^4^–10^6^ (CFU/mL) test organisms was injected at 45 °C in agar-nutrient medium and allowed to solidify. The ternary composite solution in DMSO (5 µL) and standard (Cefixime) were uniformly swabbed onto sterile paper discs, which were then placed on nutrient agar medium. The organisms were grown in triplicate plates and kept at 37 °C overnight. The diameter of the zone of inhibition was used to calculate bacterial activity. The polymer blend’s ability to resist bacteria increased with increasing the diameter of zone of inhibition. The outcomes were compared to a standard antibiotic. The negative control was DMSO, and the positive control was Cefixime (1 mg/mL).

### 4.5. Antibiofilm Assay

The ability of PMMA/ABS/CoCl_2_ composites to inhibit biofilm formation was checked against selected bacteria, *S. aureus* and *E. coli,* as a model pathogen, showing higher to lower antibacterial activity. The bacteria that form biofilm were grown in tryptic soy broth (TSB, 10 mL) and glucose (1%) for 24 h at 37 °C. A diluted bacterium strain (200 μL) was injected into 96-well microtiter plates containing 20 μg mL^−1^ ternary composites and incubated at 37 °C about 24 h. Subsequently, each well was rinsed with a pH 7.2 saline phosphate buffer and dried at ambient temperature. Crystal violet solution (200 μL, 0.1%, *w*/*v*) was added, and the mixture was kept for 30 min at 37 °C. The plates were washed and dried for 30 min. After half an hour, to get stained bound biofilms, 100 mL of absolute ethanol was added to each well. Crystal violet absorbance was measured using a Microplate Reader at 490 nm. Biofilm inhibition was measured and compared with standard, Ciprofloxacin (20 g mL^−1^) [32,33]. Percentage of inhibition was measured by

$$\%\mathrm{inhibition}=1-(\frac{\mathrm{optical}\mathrm{density}\mathrm{of}\mathrm{test}\mathrm{sample}}{\mathrm{optical}\mathrm{density}\mathrm{of}\mathrm{negative}\mathrm{control}})\times 100$$

### 4.6. Antioxidant Assay

#### 4.6.1. DPPH Free Radical Scavenging Activity

The free radical scavenging activity of the pure PMMA/ABS blend and PMMA/ABS/CoCl_2_ was determined using the DPPH method, as previously reported [34], with some minor modifications. In brief, about 3 mL of methanolic solution of DPPH (100 μM) was mixed with 1 mL of pure PMMA/ABS blend and PMMA/ABS/CoCl_2_ composites solution prepared in methanol in serial dilutions of 25, 50, 100, 200, and 400 μg/mL from stock solution. The mixture was sonicated and kept at 37 °C in a dark chamber for 30 min. After half an hour, the color of the DPPHs changed. Absorbance at 517 nm was measured on a spectrophotometer (Agilent 8453). The decrease in absorbance indicated the higher free radical inhibition activity of the mixture. The obtained findings were compared with ascorbic acid results, which was used as a reference. The formula for calculating % scavenging activity was used:

$$\%\mathrm{scavenging}\mathrm{activity}=\frac{\mathrm{absorbance}\mathrm{of}\mathrm{control}-\mathrm{absorbance}\mathrm{of}\mathrm{test}\mathrm{sample}}{\mathrm{absorbance}\mathrm{of}\mathrm{control}}\times 100$$

#### 4.6.2. ABTH^+^ Radical Scavenging Activity

The free radical inhibition activity of ABTH (2, 2-azino-bis- (3-ethylbenzothiazoline-6-sulfonic acid) of pure PMMA/ABS and PMMA/ABS/CoCl_2_ composite films were measured using the previously published method, with some modifications [35]. A methanolic solution of ABTH (7 mM) and about 2.45 mM solution of potassium persulphate were mixed and left in the dark at 37 °C for about 12 h, and absorbance was measured at 734 nm. A mixture of PMMA/ABS/CoCl_2_ (100 µL) and ABTH (1900 µL) in other vials were also left in the dark 37 °C for about 2 h. Absorbance at 734 nm was noted by a microplate reader. ABTH^+^ free radical scavenging activity was calculated as follows:

$$\%\mathrm{scavenging}\mathrm{activity}=\frac{\mathrm{absorbance}\mathrm{of}\mathrm{blank}-\mathrm{absorbance}\mathrm{of}\mathrm{test}\mathrm{composite}}{\mathrm{absorbance}\mathrm{of}\mathrm{blank}}\times 100$$

#### 4.6.3. Ferric Reducing Antioxidant Power

Ferric reducing antioxidant power (FRAP) was calculated as per the previously described method [36]. Briefly, about 20 mM of ferric chloride (FeCl_3_,) solution, 10 mM TPTZ (tripyridyl-s-triazine), and 300 mM of acetate buffer solution of pH 3.6 (1:1:10) was mixed to prepare the ferric reducing reagent. PMMA/ABS blend and PMMA/ABS/CoCl_2_ solution (100 µL) in methanol was mixed with 200 µL ferric reducing reagent by sonication, then incubated at 37 ºC for about 30 min. After half an hour, absorbance was noted at 590 nm. FRAP was calculated as µM of FeSO_4_/g of dry PMMA/ABS/CoCl_2_. Ferrous sulphate (FeSO_4_) served as a control.

## 5. Conclusions

Pure PMMA/ABS films decorated with CoCl_2_ (≤10 wt) were prepared by the solution casting technique. Structural characterization was conducted using Fourier transform infrared (FT-IR), powder X-ray diffraction, and thermal and mechanical studies. The FT-IR analysis confirmed the interaction of the PMMA and ABS blend with CoCl_2_. The degree of crystallinity of was assessed by powder XRD and measurement of the area under the peaks of PMMA, ABS, and PMMA/ABS/CoCl_2_. The area under the peaks increased as CoCl_2_ content increased, indicating a uniform mixing of CoCl_2_ with the PMMA/ABS blend and shifting the crystallinity towards a semi-crystalline nature, producing more defects in composites. The results depicted that the increase in the amount of CoCl_2_ in the PMMA/ABS blend resulted in a higher arrangement and greater thermal stability because of lower thermal motion as compared to pure PMMA/ABS films because of the predominance of random macromolecular chain scission in polymeric matrices. The mechanical properties of a PMMA/ABS/CoCl_2_ composite depends on the composition. The addition of cobalt chloride may also improve the overall mechanical properties due to the interfacial interaction caused by a solid-state coordination reaction between the nitrile groups (CN) of ABS and cobalt chloride, resulting in an increase in mechanical properties. The materials were also tested for antibacterial, antibiofilm, and antioxidant properties and showed promising results. These findings demonstrated that PMMA/ABS/CoCl_2_ films with enhanced thermomechanical, potential antibacterial, and free radical scavenging activities can be used for high-quality plastics and in a variety of pharmaceutical applications.

## Figures and Tables

**Table 1:** Mechanical properties of the PMMA/ABS blend with different contents of cobalt chloride.

CoCl_2_ (%)	PMMA/ABS Conc. (%)	Impact Strength (J.mol^−1^)	Tensile Strength (MPa)	Elastic Modulus (GPa)	Elongation at Break (%)
0	80:20	35	16.5	3.27	1.21
1.0		26	16.1	3.13	1.41
2.5		36	15	2.88	1.53
5.0		39	14	2.81	1.79
7.5		43	12	2.58	2.15
10.0		47	11	2.54	2.62

**Table 2:** Antibacterial data of a PMMA/ABS/nCoCl_2_.

Composite Materials	Zone of Inhibition (mm)					
	*S. aureus*	*S.* *pyogenes*	*B. subtilis*	*E. coli*	*S. typhi*	*P. aeruginosa*
PMMA/ABS blend	20 ± 1.5	14 ± 1.5	18 ± 1.5	19 ± 1.15	13 ± 1.25	17 ± 1.15
Blend + 2.5% CoCl_2_	24 ± 1.85	19 ± 1.35	23 ± 1.25	21 ± 1.50	19 ± 1.35	-
Blend + 5% CoCl_2_	26 ± 1.45	25 ± 1.50	26 ± 1.45	23 ± 1.35	26 ± 1.25	-
Blend + 7.5% CoCl_2_	28 ± 1.50	27 ± 1.20	31 ± 1.50	22 ± 1.25	29 ± 1.35	28 ± 1.50
Blend + 10% CoCl_2_	31 ± 1.40	28 ± 1.30	30 ± 1.25	20 ± 1.45	31 ± 1.25	29 ± 1.05
Cefixime	33 ± 1.5	31 ± 1.0	35 ± 1.15	29 ± 0.5	36 ± 1.0	31 ± 2.25

*n* = 2.5–10% CoCl_2_, ± SD, *p* ˂ 0.05; control vs. treated; 5–10 mm zone of inhibition = activity present, 11–25 mm zone of inhibition = moderate activity; 26–40 mm zone of inhibition = strong activity.

**Table 3:** Free radical scavenging data of PMMA/ABS films with 2.5–10% cobalt chloride.

Composite Materials	Antioxidant Activity		
	DPPH (%)	ABTS (%)	FRAP (%)
PMMA/ABS blend	55 ± 1.25	27 ± 2.15	0.15
Blend + 2.5% CoCl_2_	59 ± 1.5	30 ± 2.25	0.19
Blend + 5% CoCl_2_	66 ± 1.5	44 ± 1.50	0.64
Blend + 7.5% CoCl_2_	78 ± 2.0	58 ± 1.25	0.78
Blend + 10% CoCl_2_	89 ± 1.0	65 ± 2.10	0.94

## Data Availability

Not applicable.

## Sample Availability

Samples of the compounds are available from the authors.
